# Long non-coding RNA FTX predicts a poor prognosis of human cancers: a meta-analysis

**DOI:** 10.1042/BSR20203995

**Published:** 2021-01-14

**Authors:** Weiwei Chen, Yuting Li, Liliangzi Guo, Chenxing Zhang, Shaohui Tang

**Affiliations:** 1 Department of Gastroenterology, The First Affiliated Hospital, Jinan University, Guangzhou, P. R. China; 2 Department of Gastroenterology, The First People's Hospital of Zunyi, The Third Affiliated Hospital of Zunyi Medical University, Zunyi, P. R. China

**Keywords:** Cancer, Long non-coding RNA FTX, Meta-analysis, Prognosis

## Abstract

Background: Several studies have assessed the relationship between long non-coding RNA five prime to Xist (FTX) expression, clinicopathological features, and survival outcomes in patients with cancer with conflicting results. This meta-analysis synthesized existing data to clarify the association between FTX with cancer prognosis.

Methods: PubMed, Embase, Cochrane library, Web of Science, Chinese CNKI, and the Chinese WanFang databases were used to search for relevant studies. The role of FTX in cancers was evaluated by pooled odds ratios (ORs) and hazard ratios (HRs) with 95% confidence intervals (CIs).

Results: Eleven studies comprising 1210 participants including colorectal cancer (CRC), hepatocellular carcinoma (HCC), gastric cancer (GC), renal cell carcinoma (RCC), osteosarcoma (OSC), and glioma were enrolled in this analysis. The meta-analysis showed that high FTX expression was significantly associated with several clinicopathological characteristics, including lymph node metastasis in patients with CRC, GC, HCC, and RCC, distant metastasis in patients with CRC, GC, HCC, and OSC, larger tumor size in patients with CRC, GC, HCC, RCC, and OSC, and subsequently TNM/clinical stage in patients with CRC, GC, HCC, OSC, and glioma. The pooled results from the survival analysis revealed a significant correlation between high FTX expression and shorter OS in patients with HCC, CRC, GC, OSC, and glioma. Further, FTX overexpression could be an independent predictive marker for shorter OS in patients with CRC, HCC, OSC, and glioma.

Conclusions: FTX may be a potential oncogene, with high FTX expression being associated with a poorer prognosis in patients with CRC, HCC, OSC, and glioma.

## Introduction

The latest cancer statistics indicate that cancer is the leading cause of human death both in the world and in China [1,2], which results in serious economic burden. Modern treatments for cancer including surgery, adjuvant therapy, and supportive therapy have witnessed a dramatic improvement in recent years. However, many patients are at an advanced stage of cancer at the time of diagnosis, which leads to a poor prognosis.

Long non-coding RNAs (lncRNAs) are RNA molecules comprising a transcription length of more than 200 nucleotides and lack of protein-coding capacity [3]. LncRNAs were previously misunderstood as being the noise of genome transcription with no biological function [4]. However, growing evidence has revealed that lncRNAs are involved in a wide range of biological processes, such as chromosome modification, genome modification, transcriptional activation, transcriptional interference, and other processes [5]. Recent studies have shown that lncRNAs are found to be crucial in regulating cellular processes, such as cell cycle, growth, and apoptosis, which ensure homeostasis [6]. A large number of lncRNAs are associated with various types of cancer, which may exhibit tumor-suppressive and promoting (oncogenic) functions, and therefore they hold strong potential as biomarkers for the early diagnosis and prognostic prediction of cancers, and thus, also as targets of molecular targeted therapy [7].

X chromosome inactivation (XCI), the transcriptional silencing of one X chromosome that takes place in female mammals to compensate for X-linked gene dosage imbalance, is controlled and regulated by a cis-acting region on the X chromosome termed the X-inactivation center(Xic). Xist is a lncRNA gene that is the master control point in XCI. LncRNA five prime to Xist (FTX) is a highly conserved transcript of 2300 nucleotides encoded by the FTX gene, which is identified as an activator of Xist in mouse embryonic stem cells and a novel non-coding RNA involved in XCI [8]. Increasing studies have shown that FTX is related to the clinicopathological features and prognosis of patients with cancer, including colorectal cancer (CRC) [9], osteosarcoma (OSC) [10], gastric cancer (GC) [11], and renal cell carcinoma (RCC) [12]. However, these studies were limited by sample size and had opposite outcomes. For example, Liu et al. [13] indicated that FTX was significantly down-regulated in hepatocellular carcinoma (HCC) tissues compared with paired adjacent liver tissues, and the high FTX group exhibited better tumor differentiation, intact tumor encapsulation, smaller tumors, and a better overall survival. Jin et al. reported similar results, FTX expression was down‐regulated in non–small‐cell lung cancer (NSCLC) clinical tissue samples and cell lines, while ectopic expression of FTX inhibited proliferation and metastasis of lung cancer cells *in vitro* and *in vivo* [14]. In contrast, Li et al. [15] revealed that compared with paired adjacent liver tissues, HCC tissues had higher FTX expression that was significantly associated with tumor differentiation and metastasis, and predicted poor prognosis. Zhang et al. found that FTX was significantly up-regulated in gliomas, and up-regulation of FTX significantly promoted proliferation and invasion of glioma cells [16]. Therefore, it is necessary to conduct a comprehensive meta-analysis of the existing data to assess the correlation between FTX and clinicopathological features and survival outcomes in patients with cancer.

## Materials and methods

### Search strategy and study selection

The study protocol was registered with PROSPERO (registration number: CRD42020199238) before the study began. A systematic literature search was conducted in PubMed, Embase, Cochrane library, Web of Science, Chinese CNKI, and the Chinese WanFang databases from their inception through August 1, 2020.The keywords for searches were ‘long non-coding RNA FTX’ OR ‘LncRNA FTX’ AND ‘cancers’ or ‘neoplasm’ or ‘tumors’. The citation lists of relevant studies were also rigorously screened.

Studies that met the following criteria were included in the analysis: (1) the expression of FTX in patients with cancers was examined in tumor tissues and assessed using qRT-PCR; (2) the results provided survival information including overall survival (OS), disease-free survival (DFS), progression-free survival (PFS), and recurrence-free survival (RFS); (3) hazard ratios (HRs) for survival outcomes were provided or could be calculated from survival curves; and (4) the most recent paper was selected in cases of a repeat study.

The exclusion criteria for this study were as follows: (1) laboratory articles, reviews, case reports, letters, editorials, and conference reports; (2) non-human subject studies; and (3) inability to calculate HR based on the data provided.

### Data extraction and quality assessment

The full texts of potentially eligible articles were scrutinized independently by two authors (W.W.C. and Y.T.L.). Disagreements were resolved by consensus. The extracted data included the first author’s name, the year of publication, country, number of cases, type of cancer, clinicopathological features, and survival outcome. The Newcastle–Ottawa scale (NOS) criteria [17] were used to assess the quality of the studies. When the NOS score was ≥6, the article was considered to be of high-quality. An NOS score < 6 was indicative of a low-quality study.

### Statistical analysis

All the statistical data were analyzed using STATA 14.2 software. Pooled odds ratios (ORs) with 95% confidence intervals (CIs) were calculated to assess the relationship between high FTX expression and clinicopathological characteristics that included sex (male vs. female), tumor size (larger size vs. smaller size), differentiation (low vs. high-moderate), lymph node metastasis (yes vs. no), distant metastasis (yes vs. no), and TNM/clinical stage (III+IV vs. I+II). HRs with 95% CIs were calculated to assess the correlation between the overexpression of FTX and survival outcome. HRs and 95% CIs analyzed by Cox regression analysis were extracted directly from publications. If HRs and 95% CIs analyzed by the Kaplan–Meier method could not be obtained, the software Engauge Digitizer and published methods [18] were used to extract the survival data from a Kaplan–Meier curve in related articles. Heterogeneity was assessed by the *I^2^* test and *Q* test, and the random effects model was used if the *I^2^*> 50%, otherwise, a fixed effects model was used. The potential publication bias was assessed by Begg’s funnel analysis. *P*<0.05 was considered to be statistically significant. Sensitivity analysis was used to calculate the source of heterogeneity and stability of results.

## Results

### Study identification and characteristics

**Figure 1:**
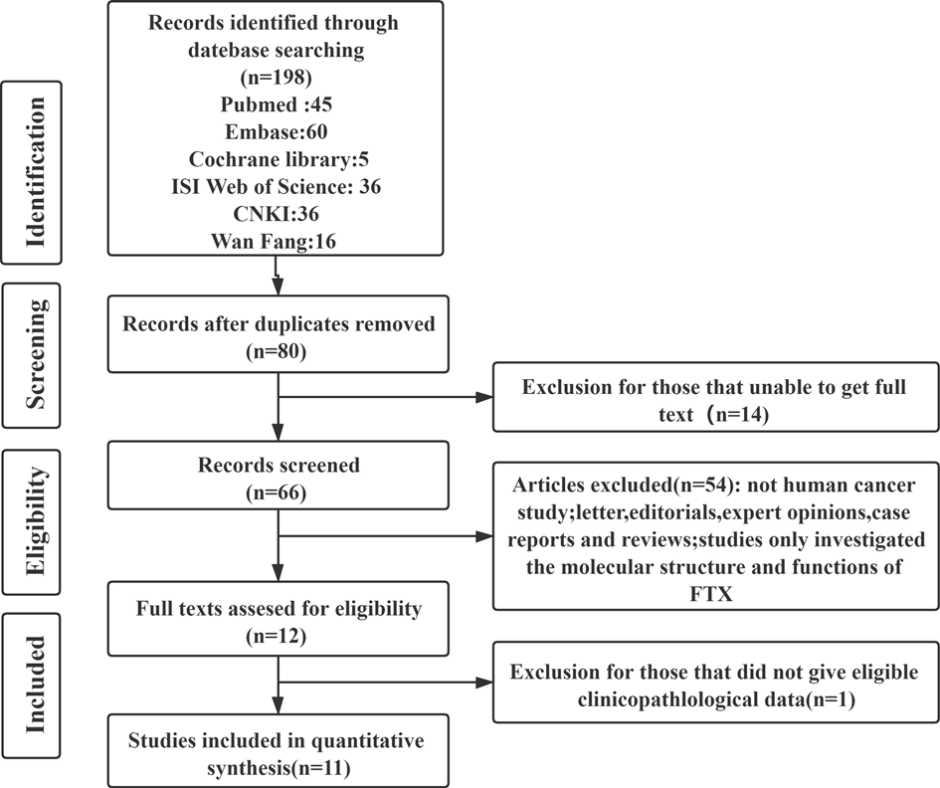
Flow diagram of study

As shown by the search flow diagram (Figure 1), a total of 11 studies [9–13,15,19–23] with 1210 patients were enrolled in this meta-analysis satisfied the inclusion criteria, including 3 studies on CRC, 3 studies on HCC, 2 studies on GC, and 1 study each on RCC, OSC, and glioma. The included studies were published from 2015 to 2020, and sample sizes ranged from 30 to 187 patients. All of included studies were from China. Nine studies were published in English and two in Chinese. All of included studies received scores ≥6 in methodological assessments, which meant they had high quality. The clinicopathological characteristics and survival information obtained from these studies are summarized in Tables 1 and 2.

**Table 1:** Characteristics of studies included in the meta-analysis

Study	Year	Country	Cancer type	Detection method	Sample size	LncRNA FTX expression														Survival information	NOS score
						High							Low								
						Total	LNM	DM	HS	LD	Larger TS	M	Total	LNM	DM	HS	LD	Larger TS	M		
Zhao et al. [9]	2020	China	CRC	RT-qPCR	30	15	9	11	10	NA	10	9	15	6	7	7	NA	6	7	NA	8
He et al. [12]	2017	China	RCC	RT-qPCR	150	82	63	NA	65	NA	76	42	68	30	NA	34	NA	48	37	NA	8
Liu et al. [13]	2016	China	HCC	RT-qPCR	129	64	NA	NA	16	43	45	51	65	NA	NA	16	56	57	60	OS/RFS	7
Li et al. [10]	2018	China	OSC	RT-qPCR	84	39	NA	17	23	NA	25	22	45	NA	9	14	NA	22	27	OS	9
Zhang et al. [11]	2020	China	GC	RT-qPCR	71	32	25	NA	26	19	17	17	39	21	NA	18	11	18	22	OS	7
Liang et al. [17]	2020	China	glioma	RT-qPCR	187	95	NA	NA	50	NA	NA	40	92	NA	NA	26	NA	NA	49	OS/PFS	7
Guo et al. [16]	2015	China	CRC	RT-qPCR	187	75	46	NA	44	38	NA	14	112	71	NA	60	51	NA	31	OS	6
Jiang et al. [18]	2019	China	GC	RT-qPCR	93	48	34	15	36	27	35	31	45	26	4	23	6	14	29	NA	9
Li et al. [14]	2019	China	HCC	RT-qPCR	73	37	18	18	NA	2	23	27	36	6	6	NA	12	21	20	OS/DFS	6
Yang et al. [20]	2018	China	CRC	RT-qPCR	80	40	27	25	26	12	26	27	40	15	12	17	14	17	26	OS	8
Liu et al. [21]	2016	China	HCC	RT-qPCR	126	65	NA	NA	18	32	28	50	61	NA	NA	8	12	10	49	OS/DFS	7

Abbreviations: CRC, colorectal cancer; DFS, disease-free survival; DM, distant metastasis; GC, gastric cancer; HCC, hepatocellular carcinoma; HS, high TNM/clinical stage (III/IV); Larger TS, the larger tumor size group (diameter ≥3 cm); LD, low differentiation; LNM, lymph node metastasis; M, man; OS, overall survival; NA, not available; NOS, Newcastle–Ottawa scale; OSC, osteosarcoma; PFS, progression-free survival; RCC, renal cell carcinoma; RFS, recurrence-free survival; RT-qPCR, reverse transcription quantitative real-time polymerase chain reaction.

**Table 2:** Survival data of studies included in the meta-analysis

Study	Year	Cancer type	Detection method	Sample size	Survival information				HR statistic
					Overall survival (OS)		Other survival		
					Univariate analysis	Multivariate	Univariate analysis	Multivariate	
Liu et al [13]	2016	HCC	RT-qPCR	129	0.59 (0.34–1.03) (E)	NA	0.51 (0.30–0.89) (E)^1^	NA	Survival curve
Li et al [10]	2018	OSC	RT-qPCR	84	2.636 (1.458–4.767)	2.162 (1.158–4.037)	NA	NA	Data in paper
Zhang et al [11]	2020	GC	RT-qPCR	71	2.07 (1.33–3.24) (E)	NA	NA	NA	Survival curve
Liang et al [17]	2020	glioma	RT-qPCR	187	1.36 (0.90–2.07) (E)	5.358 (1.775–8.569)	1.69 (1.15–2.49) (E)^2^	6.323 (2.213–9.934)^2^	Both of two
Guo et al [16]	2015	CRC	RT-qPCR	187	151 (0.98–2.32) (E)	2.371 (1.42–2.739)	NA	NA	Both of two
Li et al [14]	2019	HCC	RT-qPCR	73	12.456 (1.607–96.543)	10.376 (1.284–83.836)	7.484 (3.398–16.486)^3^	6.411 (2.859–14.375)^3^	Data in paper
Yang et al [20]	2018	CRC	RT-qPCR	80	1.32 (0.80–2.17) (E)	NA	NA	NA	Survival curve
Liu et al [21]	2016	HCC	RT-qPCR	126	1.92 (1.23–2.92) (E)	NA	2.08 (1.32–3.27) (E)^3^	NA	Survival curve

1 Recurrence-free survival.

2 Progression-free survival.

3 Disease-free survival. Abbreviations: CRC, colorectal cancer; E, survival data were obtained by using software Engauge Digitizer; GC, gastric cancer; HCC, hepatocellular carcinoma; HR, hazard ratio; NA, not available; OSC, osteosarcoma; RCC, renal cell carcinoma; RT-qPCR, reverse transcription quantitative real-time polymerase chain reaction.

### FTX expression and clinicopathological characteristics

**Figure 2:**
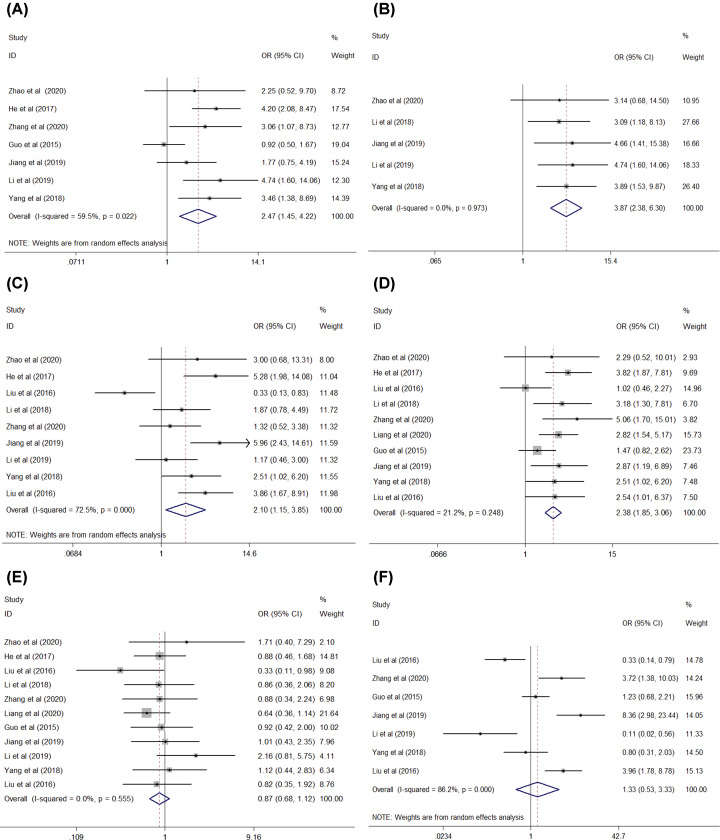
Forest plot of studies evaluating the relationship between FTX expression and clinicopathological features (**A**) Lymph node metastasis, (**B**) distant metastasis, (**C**) tumor size, (**D**) TNM/clinical stage, (**E**) gender, (**F**) differentiation.

The pooled ORs with 95% CI are shown in Table 3. The results revealed that elevated FTX expression was significantly associated with lymph node metastasis (yes vs. no: OR = 2.47, 95% CI [1.45, 4.22], *P*=0.001, Figure 2A) in seven studies (three studies on CRC, two studies on GC, and one study each on HCC and RCC) [9,11,12,15,20–22], distant metastasis (yes vs. no: OR = 3.87, 95% CI [2.38, 6.30], *P*<0.001, Figure 2B) in five studies (two studies on CRC and one study each on GC, HCC, and OSC) [9,10,15,21,22], bigger tumor size (larger vs. smaller: OR = 2.10, 95% CI [1.15, 3.85], *P*=0.016, Figure 2C) in nine studies (two studies each on CRC and GC, three studies on HCC, and one study each on RCC and OSC) [9–13,15,21–23], and subsequently on TNM/clinical stage (III+IV vs. I+II : OR = 2.38, 95% CI [1.85, 3.06], *P*<0.001, Figure 2D in ten studies (three studies on CRC, two studies each on GC and HCC, one study each on RCC, OSC, glioma) [9–13,19–23]. The overexpression of FTX was not associated with sex (male vs. female: OR = 0.87, 95% CI [0.68, 1.12], *P*=0.271, Figure 2E) in all the 11 aforementioned studies and differentiation (low vs. high and moderate: OR = 1.33, 95% CI [0.53, 3.33], *P*=0.546, Figure 2F) in seven studies (two studies on CRC, three studies on GC, and two studies on HCC) [11,13,15,20–23]. The findings suggest that high expression of FTX is associated with clinicopathological parameters of the aforementioned cancers.

**Table 3:** LncRNA FTX clinicopathological features for cancers

Clinicopathological features	No. of studies	No. of patients	Pooled OR (95% CI)	*P* Het	*I*^2^ (%)	*P* value	Model used
Gender	11	1210	0.87 (0.68–1.12)	0.555	0	0.271	Fixed
Lymph node metastasis	7	684	2.47(1.45–4.22)	0.002	59.5	0.001	Random
Distant metastasis	5	360	3.87(2.38–6.30)	0.973	0	<0.001	Fixed
Tumor size	9	836	2.10(1.15–3.85)	0	72.5	0.016	Random
Differentiation	7	759	1.33 (0.53–3.33)	0	86.2	0.546	Random
TNM/clinical stage	10	1137	2.38 (1.85–3.06)	0.248	21.2	<0.001	Fixed

Abbreviations: CI, confidence interval; Fixed, fixed effects model; OR, odds ratio; Random, random effects model.

### FTX expression and survival outcomes

**Figure 3:**
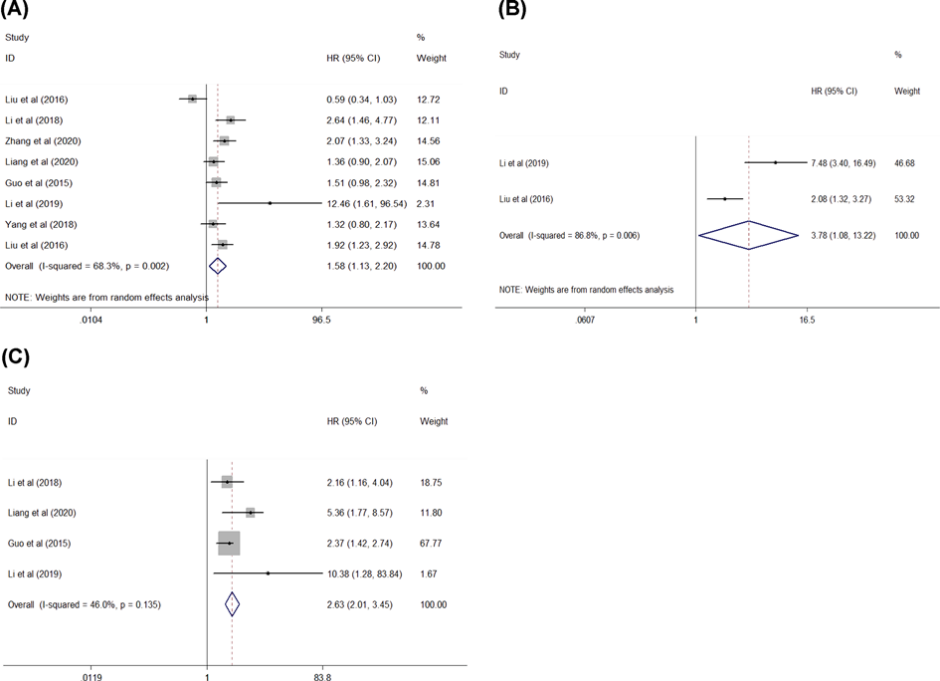
Forest plot of studies evaluating the relationship between FTX expression and the survival rate (**A**) Overall survival (OS) rate, (**B**) disease-free survival (DFS) rate, (**C**) independent predictive factor for OS.

A meta-analysis was conducted using the data from survival analysis using the Kaplan–Meier method in eight studies (three studies on HCC, two studies on CRC, and one study each on GC, OSC, and glioma) [10,11,13,15,19,20,22,23]. The pooled HRs for OS was extracted from eight heterogeneous studies (*I^2^*= 68.3%, *P*=0.002) comprising 937 patients, and the results showed a significant correlation between high FTX expression and a shorter OS (high vs. low: HR = 1.58, 95% CI [1.13, 2.20], *P* = 0.007, Figure 3A). In addition, the two heterogeneous studies [15,23] (*I^2^* = 86.8%, *P* = 0.006) with 199 patients with HCC were included, and the pooled results indicated that high FTX expression was related to a shorter DFS (high vs. low: HR = 3.78, 95% CI = [1.08, 13.22], *P*=0.037, Figure 3B).

Another meta-analysis was conducted using data from the survival analysis in a multivariate Cox regression model that included four studies (one study each on CRC, HCC, OSC, and glioma) and a total of 531 patients [10,15,19,20]. The role of FTX as an independent predictive factor for OS in patients with cancer was assessed. The pooled results showed that higher FTX expression was an independent prognostic factor for a shorter OS of patients with the aforementioned four cancers (HR = 2.63, 95% CI [2.01, 3.45], *P*<0.001, Figure 3C).

### Sensitivity analysis

We performed sensitivity analyses to evaluate the robustness of the prognostic model. The results (Supplementary Tables S1 and S2) were not significantly altered when any individual study was removed, demonstrating that the results were reliable.

### Publication bias

**Figure 4:**
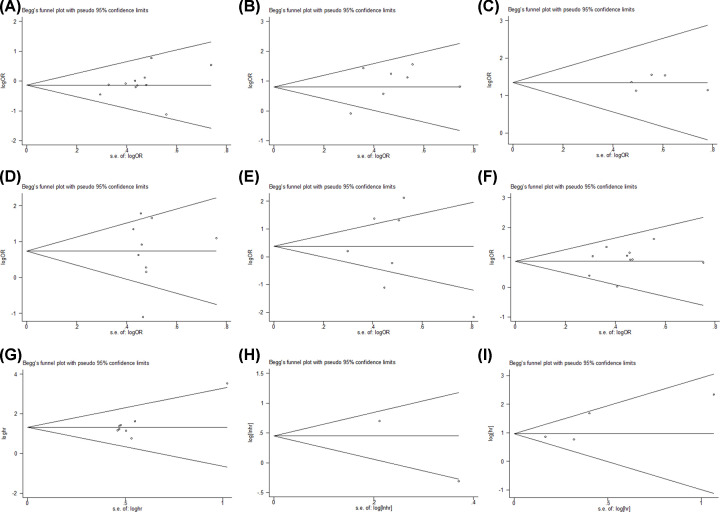
Begg’s publication bias plots for FTX related studies (**A**) Gender, (**B**) lymph node metastasis, (**C**) distant metastasis, (**D**) tumor size, (**E**) differentiation, (**F**) TNM/clinical stage, (**G**) overall survival (OS) rate, (**H**) disease-free survival (DFS) rate, and (**I**) independent predictive factor for OS.

Begg’s funnel analysis was conducted to evaluate the publication bias. As shown in Figure 4, there was no publication bias for sex (*P*=0.276), lymph node metastasis (*P*=0.764), distant metastasis (*P*=1.0), tumor size (*P*=0.754), differentiation (*P*=1.0), TNM/clinical stage (*P*=0.858), OS (*P*=0.174) or DFS (*P*=1.0), and the model was an independent factor for OS (*P*=0.308).

## Discussion

In the 1980s, the first regulatory non-coding RNA (ncRNA) was identified in bacteria, and subsequently, a large number of ncRNAs have been found in most eukaryotic organisms [24]. LncRNAs belong to this class of ncRNAs and were initially dismissed as being merely transcriptional ‘noise’ [25]. However, emerging evidence has revealed that lncRNAs are associated with the development of cancer by regulating various cellular functions, including proliferation, migration, and DNA stability [26]. Because of the tissue-specific characteristics of lncRNAs, they may become the next generation of biomarkers or targets for human cancer [27].

FTX is a member of the lncRNA family. In recent years, changes in FTX expression in different types of cancers and the influence of FTX expression in cancer have attracted much attention. Nonetheless, the effects of FTX expression on the development and prognosis of cancer are still controversial. Therefore, we combined all published studies investigating this issue to conduct this meta-analysis.

A total of 11 eligible studies comprising 1210 cancer patients, reporting six common cancer types, were included. Our results showed that high FTX expression was significantly associated with several clinicopathological characteristics, including lymph node metastasis in patients with CRC, GC, HCC, and RCC; distant metastasis in patients with CRC, GC, HCC, and OSC; bigger tumor size in patients with CRC, GC, HCC, RCC, and OSC; and later TNM/clinical stage in patients with CRC, GC, HCC, OSC, and glioma. However, there were no significant differences between the high and low FTX expression groups with respect to sex and tumor differentiation. Conversely, a significant association between high FTX expression and a shorter OS in patients with CRC, GC, HCC, OSC, and glioma, or a shorter DFS in patients with HCC was observed. Further meta-analysis showed that high FTX expression could be an independent predictive marker for shorter OS in patients with CRC, HCC, OSC, and glioma. Taken together, these results suggested that FTX may be a candidate oncogene, and the overexpression of FTX was associated with a poor prognosis in common solid malignant tumors, such as CRC, HCC, OSC, and glioma.

Based on evidence from previous studies, cancer promotion by FTX may involve the following mechanisms: FTX significantly promotes the proliferation and invasion of glioma cells by negatively regulating miR-342-3p [16]; FTX exerts its oncogenic role in OSC via up-regulating the expression of TXNRD1 through sponging miR-320a [28]; FTX promotes proliferation and invasion of gastric cancer via the miR-144/ZFX axis [29]; FTX functions as an oncogene to contribute to CRC progression by regulating the miR-192-5p/EIF5A2 axis [9].

Our results were similar to those of previous reports. For example, in the meta-analysis investigating the lncRNA GHET-1, GHET-1 expression was closely correlated with tumor size, lymph node metastasis, distant metastasis and TNM stage, and increased GHET-1 expression may be a potential prognostic biomarker in human cancers including HCC, GC,OSC, breast cancer, baldder cancer, cervical cancer, NSCLC, and esophageal cancer (ESCC) [30]. Zhu et al. [31] conducted a meta-analysis which demonstrated that elevated lncRNA XIST expression predicted poor OS, poor DFS, larger tumor size, increased distant metastasis and advanced tumor stage, suggesting that high lncRNA XIST expression may serve as a novel biomarker for poor prognosis and metastasis in cancers (CRC, GC, HCC, ESCC, pancreatic cancer [PC], nasopharyngeal carcinoma [NPC], and glioma).

Nevertheless, several limitations existed in this meta-analysis. The main limitation of the present study was the small number of included studies and patients (11 studies and 1210 patients), thus further subgroup analysis could not be performed stratifying patients by age group and tumor site. Subgroup analysis was not carried out for different types of tumors to reduce the heterogeneity caused by different types of tumors. Second, all of the studies were from China, so the results may only be applicable to the Chinese populations. Large-scale and well-designed studies are still needed to verify the clinical value of FTX in different ethnicities. Third, some studies did not provide HRs directly, and an available software was used to calculate the HRs values, which may have introduced errors. Fourth, the included studies had different criteria for the classification of FTX expression, which may have affected the heterogeneity of the meta-analysis.

## Conclusions

FTX may be a potential oncogene, and its high expression be associated with a poor prognosis in patients with CRC, HCC, OSC, and glioma. Additional high-quality studies involving large numbers of patients are needed to confirm the findings.

## Supplementary Material

Supplementary Tables S1-S2

## Data Availability

The data used to support the findings of this study are included within the article. The primary data used to support the findings of this study are available from the corresponding author upon request.

## Abbreviations

CI: confidence interval
CRC: colorectal cancer
DFS: disease-free survival
DM: distant metastasis
ESCC: esophageal cancer
GC: gastric cancer
HCC: hepatocellular carcinoma
HR: hazard ratio
NPC: nasopharyngeal carcinoma
OR: odds ratio
OS: overall survival
OSC: osteosarcoma
RCC: renal cell carcinoma
RT-qPCR: reverse transcription quantitative real-time polymerase chain reaction
