# Supplementary material for: Long non-coding RNA FTX predicts a poor prognosis of human cancers: a meta-analysis
Source: Biosci Rep. 2021 Jan 14;41(1):BSR20203995. doi: 10.1042/BSR20203995 (PMC7809557; doi:10.1042/BSR20203995)
Supplement: Supplementary Tables S1-S2 [file BSR-2020-3995_supp.pdf]

Supplementary table 1. The sensitivity analysis of the relationship between FTX and OS

Excluded studies	No. of included studies	I^2	HR	95%CI	*P*
no	8	68.30%	1.58[a]	(1.13-2.20)	0.007
Liu et al,2016	7	34.40%	1.73[b]	(1.43-2.08)	＜0.001
Li et al,2018	7	67.90%	1.47a	(1.04-2.08)	0.03
Zhang et al,2020	7	70.20%	1.51[a]	(1.04-2.20)	0.031
Liang et al,2020	7	72.30%	1.63[a]	(1.10-2.42)	0.015
Guo et al,2015	7	72.80%	1.60[a]	(1.08-2.38)	0.019
Li et al,2019	7	66.80%	1.50[a]	(1.10-2.05)	0.01
Yang et al,2018	7	72.30%	1.63[a]	(1.11-2.40)	0.012
Liu et al,2016	7	71.30%	1.54[a]	(1.04-2.26)	0.029

a: random effects model; b:fixed effects model

Supplementary table 2. The sensitivity analysis of independent prognostic value of FTX in cancers

Excluded studies	No. of included studies	I^2	HR	95%CI	*P*
no	4	46%	2.63[b]	(2.01-3.45)	＜0.001
Li et al,2018	3	60.70%	3.73[a]	(1.74-8.00)	0.001
Liang et al,2020	3	0%	2.39[b]	(1.79-3.19)	＜0.001
Guo et al,2015	3	54.30%	3.79[a]	(1.68-8.58)	0.001
Li et al,2019	3	48.30%	2.57[b]	(1.96-3.37)	＜0.001

a: random effects model; b:fixed effects model
